# Exploring the role of advanced MRI in understanding glioblastoma biology: A scoping review protocol

**DOI:** 10.1016/j.mex.2026.103984

**Published:** 2026-06-02

**Authors:** James Brown-Miles, Oun Al-iedani, Peter Greer, Michael Fay, Hubert Hondermarck, Saadallah Ramadan

**Affiliations:** a School of Biomedical Sciences and Pharmacy, College of Health, Medicine and Wellbeing, University of Newcastle, Callaghan, New South Wales, Australia; b Hunter Medical Research Institute, New Lambton Heights, New South Wales, Australia; c Mark Hughes Foundation Centre for Brain Cancer Research, University of Newcastle, New Lambton Heights, New South Wales, Australia; d NSW Regional Cancer Research Network, NSW Regional Health Partners, Cancer Institute NSW, New Lambton Heights, New South Wales, Australia; e School of Information and Physical Sciences, College of Engineering, Science and Environment, University of Newcastle, Callaghan, New South Wales, Australia; f Department of Radiation-Oncology, Calvary Mater Newcastle, Waratah, New South Wales, Australia; g School of Medicine and Public Health, College of Health, Medicine and Wellbeing, University of Newcastle, Callaghan, New South Wales, Australia; h GenesisCare, Gateshead, New South Wales, Australia

**Keywords:** Scoping review protocol, 2021 who classification, Magnetic resonance imaging, MRI, Glioblastoma, GBM, Preoperative, Brain Cancer, Neuro-oncology

## Abstract

Advanced magnetic resonance imaging (MRI) offers unique opportunities to explore the biological complexity of glioblastoma, the most aggressive brain tumour. The 2021 World Health Organization reclassification of glioblastoma has obscured the interpretation of prior imaging research, necessitating a focussed synthesis to align it with current guidelines. This scoping review protocol aims to systematically map evidence from adult, preoperative, isocitrate dehydrogenase-wildtype glioblastoma to answer the question, "can advanced MRI help understand the biology of glioblastoma?" It will focus on peer-reviewed studies investigating associations between advanced MRI and tumour characteristics, including correlations with histopathological/molecular markers. Five databases will be searched for primary sources using advanced MRI, such as magnetic resonance spectroscopy, diffusion-, perfusion-, chemical exchange saturation transfer- and susceptibility-weighted imaging, to image glioblastoma. Findings will be stratified by biological domains, such as molecular factors, vascularisation and metabolism.

• Databases: PubMed, Scopus, Cochrane, EBSCO, Embase.
• Methodology: Preferred Reporting Items for Systematic reviews and Meta-Analyses extension for Scoping Reviews framework and Joanna Briggs Institute guidance.
• Outcome: Evidence map and narrative synthesis of biological insights derived from advanced MRI, aligning literature with the current classification and highlighting methodological gaps and priorities for validation and clinical translation to guide future study design and standardisation efforts.

## Graphical abstract

**Figure:**
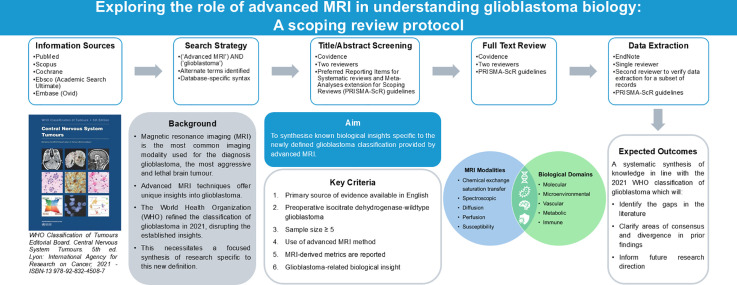

## Specifications table

**Table:** 

**Subject area**	Neuroscience
**More specific subject area**	Neuro-oncology
**Name of your protocol**	Exploring the role of advanced MRI in understanding glioblastoma biology: A scoping review protocol
**Reagents/tools**	Not applicable
**Experimental design**	Scoping review protocol
**Trial registration**	Not applicable
**Ethics**	This scoping review protocol does not involve human participants, animal experiments or data collected from social media platforms. It will use data from published research articles; hence, ethical approval and informed consent are not required.
**Value of the Protocol**	• Glioblastoma was reclassified in 2021 by the World Health Organization, mandating a new synthesis of knowledge to reduce the heterogeneity of past findings • This scoping review protocol encourages reproducibility and transparency in a systematic methodology • The resulting review will inform the future direction of research

## Background

Magnetic resonance imaging (MRI) is routinely implemented in both the clinical and research setting when investigating glioblastoma [1]. However, the typical usage of conventional MRI methods – those based only on the relaxation times, including T1-weighted and T2-weighted sequences - is limited, as it is unable to detect the occult infiltration of tumour cells from the foci of the tumour [2]. This limitation has significant implications on the outcomes of treatment, since conventional MRI is used for surgical planning, guiding radiotherapy and monitoring for progression. Therefore, there is a need to further develop and validate more advanced MRI methods - such as diffusion-weighted imaging (DWI), perfusion-weighted imaging (PWI), magnetic resonance spectroscopy (MRS), and susceptibility-based methods - for the improved diagnosis and prognostication of glioblastoma. Advanced MRI techniques are capable of measuring unique properties; DWI obtains a signal based on the Brownian motion of water to provide information about the microstructure of the tumour, including its cellularity [3], while diffusion tensor imaging (DTI), an extension of DWI, is sensitive to disruptions of white matter tracts [4]. PWI provides information about the vascularity of the tumour via the flow and/or volume of cerebral blood, and is often used to confirm a diagnosis [1]. MRS measures the chemical shift of hydrogen in metabolites endogenous to the brain providing measures of their concentrations [5]. Susceptibility-weighted imaging (SWI) and quantitative susceptibility mapping (QSM) are able to detect iron and calcification by measuring their effects on the magnetic field [6]. It is important to note that the interpretation of imaging–derived associations is shaped by well‑recognised methodological challenges [7]. Reproducibility is affected by hardware and protocol differences, as well as post‑processing choices. Standardisation has also progressed unevenly across modalities [6,[8], [9], [10], [11]], and uncertainty quantification is variably reported – both are factors that may amplify heterogeneity and complicate biological interpretation, as such, our protocol documents and contextualises findings accordingly in the synthesis.

Glioblastoma is notoriously heterogenous, and in 2021 the World Health Organization (WHO) updated its classification, incorporating both histological and molecular characteristics in the diagnostic criteria [12]. This update separated ‘glioblastoma multiforme’ into two distinct tumour types, WHO grade 4 glioblastoma isocitrate dehydrogenase (IDH)-wildtype and WHO grade 4 astrocytoma IDH-mutant. This shift fundamentally changes how previous imaging studies should be interpreted, as many were conducted under the older histology-based framework, grouping biologically distinct tumours together. Imaging biomarkers reported in those studies may therefore reflect mixed molecular populations, limiting their relevance to current diagnostic categories and creating an urgent need for re-evaluation. The reclassification recognises the prognostic differences between these types, enabling the definition of more homogenous cohorts both in the clinical and research settings [13]. This will reduce confounding and allow a more focussed approach in future studies. Hence, a new synthesis that reconciles the diagnostic criteria of the two systems, as used to describe research cohorts, is required. Accordingly, this protocol aims to systematically map existing evidence to achieve a focussed synthesis of advanced MRI research on adult, preoperative, IDH-wildtype glioblastoma. This is made possible by the approximate equivalence of historical primary/de novo glioblastomas to the 2021 WHO classification of glioblastoma IDH-wildtype, while the historical secondary glioblastomas equate to grade 4 IDH-mutant gliomas [[13], [14], [15], [16]]. The implications for previous imaging studies include the need to reinterpret reported imaging correlates in light of molecular stratification and to determine whether observed patterns remain valid within these biologically defined subgroups. This synthesis will ensure that prior studies remain clinically relevant and up to date. Herein, ‘glioblastoma’ will be used in line with the 2021 classification.

While the IDH mutation status is a key molecular marker for diagnosis, it is not the only one to play a significant prognostic role in the management of glioblastoma [17]. Other key factors include the O6-methylguanine-DNA methyltransferase promoter (MGMTp) methylation status [18,19], epidermal growth factor receptor (EGFR) amplification status [20], and telomerase reverse transcriptase promoter (TERTp) mutations [21], which also impact treatment response and prognosis. Numerous exploratory studies have investigated the potential correlation between advanced MRI metrics and relevant biological factors [[22], [23], [24], [25]], suggesting opportunities for the non-invasive characterisation of glioblastoma. Molecular alterations shape tumour biology—metabolism, vascularity, and invasiveness—which in turn influence measurable MRI features. Understanding these links enables imaging to serve as a surrogate for key biomarkers, supporting precision diagnostics and treatment planning. One such example in gliomas is the use of MRS to detect the presence of 2-hydroxyglutarate (2HG), an oncometabolite that is only produced in IDH-mutant brain tumours [26,27], thereby acting as an imaging-based biomarker that preoperatively informs a key diagnostic criterion. Further potential likely lies in the relationship between molecular alterations that influence the (neo)vasculature, such as EGFR amplification, and PWI, however, exact relationships will be elucidated upon completion of this review.

The 2021 WHO update complicates interpretation of prior studies because older research grouped tumours based on histology alone, whereas the new framework integrates molecular markers such as IDH mutation and 1p/19q codeletion as defining criteria. As a result, imaging features previously attributed to “glioblastoma” may actually reflect mixed molecular populations with different biology and prognosis. This means that conclusions drawn under the old classification cannot be directly applied to current practice without re-evaluation and stratification by molecular subtype. Existing reviews [7,[28], [29], [30], [31], [32], [33], [34], [35], [36], [37], [38], [39], [40]] focus on either the technical aspects of imaging modalities, specific applications of deep-learning models / radiomics, or broad imaging biomarkers, without stratifying by molecular subtypes or aligning with the 2021 WHO classification - this limits their clinical applicability. Critically, these prior syntheses have several limitations, firstly, they do not systematically address the heterogeneity introduced by the 2021 WHO reclassification, they often combine findings across molecularly distinct tumour types. Furthermore, existing reviews have not comprehensively mapped the landscape of advanced MRI research specifically for preoperative, treatment-naïve glioblastoma, nor have they systematically identified which biological domains are under-explored or which imaging modalities show the greatest utility for specific biological questions.

This scoping review protocol was designed to address this lack of synthesis. It outlines a systematic approach to harmonise the field’s existing research in line with the 2021 WHO reclassification, to provide a comprehensive and more clinically meaningful characterisation of glioblastoma. The review will explore how advanced MRI techniques can confer a better understanding of differences between molecular subtypes of glioblastoma via synthesis of eligible imaging studies conducted in preoperative, treatment-naïve human glioblastoma cohorts. By highlighting the current state of knowledge, the review will identify any gaps that exist in the literature, clarify areas of consensus and divergence, and inform the direction of future research. Importantly, improved biological characterisation through advanced MRI has several direct clinical implications: it could guide surgical planning by more accurately delineating tumour infiltration, refine prognostication by linking imaging features to key molecular markers, and support personalised therapy selection, including radiotherapy targeting and pharmacologic strategies. Such insights may also accelerate drug development and enable more sensitive screening and monitoring, particularly during the development of new therapies within clinical trials.

The expected scientific contributions of this review include: (1) providing a comprehensive evidence map that identifies imaging-biology relationships, and informs which are well-established versus exploratory; (2) highlighting methodological gaps and inconsistencies that should be addressed in future research design; (3) facilitating translation of imaging biomarkers into clinical practice by clarifying which findings are sufficiently robust for clinical implementation versus those requiring further validation; and (4) accelerating the development of imaging-based precision diagnostics by synthesising current knowledge in a framework aligned with contemporary molecular classification systems. This synthesis will serve as a foundation for designing prospective validation studies, developing standardised imaging protocols for multi-centre trials, and identifying priority areas for advanced MRI research in glioblastoma.

## Description of protocol

### Design and research question

A scoping review was identified as the appropriate approach as it is well-suited to map a broad, complex field of research, as required by the primary research question: “can advanced MRI help understand the biology of glioblastoma?” A systematic review was rendered less appropriate, since it is designed to answer highly specific questions about intervention effectiveness, requiring homogeneous study designs and outcome measures. In contrast, the literature on advanced MRI and glioblastoma biology is inherently heterogeneous in methods, imaging metrics, and endpoints, making a scoping review better suited to map and synthesise this complexity.

To address the primary research question, we seek to answer the following six research questions (RQ):

• RQ1: What biological insights have been established using advanced MRI for glioblastoma?
• RQ2: Which advanced MRI modality shows the most utility, determined by their correlation with, or ability to predict the status of, histological and molecular markers, for elucidating biological insights into glioblastoma?
• RQ3: Which advanced MRI technologies are currently under-researched and/or under-utilised for investigating glioblastoma-related biology?
• RQ4: What aspects of glioblastoma-related biology are currently under-explored using advanced MRI techniques?
• RQ5: What are the typical limitations of studies using advanced MRI to investigate glioblastoma-related biology?
• RQ6: What are the key suggestions for improvements in future studies?

The primary objective of the planned scoping review is to synthesise the known biological insights specifically aligned with the 2021 WHO classification of glioblastoma, as derived from advanced MRI techniques. This will involve mapping the distribution and frequency of use for each MRI modality – individually and in combination – and identifying how frequently each aspect of glioblastoma biology is investigated.

### Working definition of ‘advanced MRI’

For the purpose of this protocol, ‘advanced MRI’ is operationally defined for each modality as follows:

• CEST: amide proton transfer-weighted (APTw) imaging, glucoCEST (glucose uptake),
• Diffusion: DWI, DTI, diffusion kurtosis imaging (DKI), neurite orientation dispersion and density imaging (NODDI),
• Perfusion: dynamic susceptibility contrast imaging (DSC), dynamic contrast enhanced imaging (DCE), arterial spin labelling (ASL),
• Spectroscopic: MRS,
• Susceptibility: SWI, QSM,
• Physiologic: quantitative blood oxygen level-dependent (qBOLD) oxygen-metabolism mapping, vascular architecture mapping (VAM) and
• Functional: BOLD imaging.

### Working definition of ‘biological insight’

Within this protocol, ‘biological insight’ is used to mean information pertaining to tumour biology or its impact on the wider brain/physiology, including but not limited to histological markers, molecular markers, altered metabolism, immune response and (neo)vasculature/angiogenesis. This can be by correlative, mechanistic or predictive means – all three forms of analysis will be captured for synthesis in this review, as the aim is to seek out research that links imaging features to tumour biology.

### Protocol and registration

This protocol adheres to the guidelines set out in the Preferred Reporting Items for Systematic reviews and Meta-Analyses extension for Scoping Reviews (PRISMA-ScR) framework [41] and aligns with the methodological guidance provided by the Joanna Briggs Institute (JBI) for scoping reviews [[42], [43], [44]]. This protocol has not been registered in any repository such as the open science framework (OSF: https://osf.io/) or the International prospective register of systematic reviews (PROSPERO: https://www.crd.york.ac.uk/prospero/).

### Information sources

To identify primary sources of evidence that adhere to the eligibility criteria, the following five databases will be utilised: PubMed, Scopus, Cochrane, EBSCO (Academic Search Ultimate) and Embase (Ovid). Institutional access will be utilised to conduct the searches presented in this protocol.

Automated alerts will be set up to continuously monitor newly published articles that match the initial search strategy, and these will be screened using the same eligibility criteria. These database searches will not be supplemented by reference list scanning, nor hand-searching of key journals, to preserve reproducibility and reduce selection bias.

### Search strategy

The search strategy was developed to be inclusive of all terminologies and abbreviations that have been used to describe advanced MRI technologies over time. It was structured using the Population-Concept-Context (PCC) framework and adapted to the syntax of each database, as listed in Table 1.

**Table 1:** Search strategies designed and adapted to each database.

Database	Search Strategy
PubMed	(MR[Title] OR MRI[Title/Abstract] OR "magnetic resonance"[Title/Abstract] OR "APT" OR "CEST" OR "amide proton transfer" OR "chemical exchange saturation transfer" OR "DWI" OR "DTI" OR "diffusion-weighted imag*" OR "diffusion tensor imag*" OR "SWI" OR "QSM" OR "susceptibility-weighted imag*" OR "quantitative susceptibility map*" OR "dynamic contrast enhanc*" OR "dynamic susceptibility contrast-enhanc*" OR "DKI" OR "diffusion kurtosis imag*" OR "PWI" OR "perfusion-weighted imag*" OR "arterial spin label*") AND (glioblastoma[Title/Abstract] OR GBM[Title/Abstract] OR "HGG" OR ((glioma[Title/Abstract] OR astrocytoma[Title/Abstract] OR "brain tumo*"[Title/Abstract]) AND (("grade 4″[Title/Abstract:∼8]) OR ("grade IV"[Title/Abstract:∼8]) OR "high-grade"[Title/Abstract:∼4]))) NOT ((review[pt]) OR (systematic review[pt]) OR (meta-analysis[pt]) OR (case reports[pt]))
Scopus	(TITLE-ABS-KEY (MRI) OR TITLE-ABS-KEY ("magnetic resonance") OR TITLE ("mr") OR TITLE-ABS-KEY ("amide-proton transfer") OR TITLE-ABS-KEY ("chemical exchange saturation transfer") OR TITLE-ABS-KEY ("diffusion-weighted imaging") OR TITLE-ABS-KEY ("diffusion tensor imaging") OR TITLE-ABS-KEY ("susceptibility-weighted imaging") OR TITLE-ABS-KEY ("quantitative susceptibility mapping") OR TITLE-ABS-KEY ("arterial spin labelling") OR TITLE-ABS-KEY ("diffusion kurtosis imaging") OR TITLE-ABS-KEY ("perfusion-weighted imaging")) AND (TITLE-ABS-KEY (glioblastoma) OR TITLE-ABS-KEY (GBM) OR TITLE-ABS-KEY ("high-grade glioma")) AND (SUBJAREA(MEDI) OR SUBJAREA(BIOC) OR SUBJAREA(NEUR) OR SUBJAREA(HEAL) OR SUBJAREA(CHEM) OR SUBJAREA(PHYS) OR SUBJAREA(PHAR) OR SUBJAREA(IMMU) OR SUBJAREA(AGRI)) AND (KEY (adult) OR KEY (human) OR KEY(humans) OR KEY("in vivo study")) AND NOT KEY ("human tissue") AND NOT KEY ("case report") AND NOT KEY ("child") AND NOT KEY ("nonhuman") AND NOT KEY ("animals") AND NOT KEY ("animal experiment") AND NOT KEY ("animal model") AND NOT KEY ("mouse") AND NOT KEY ("animal") AND NOT KEY ("cell line, tumor") AND NOT KEY ("animal tissue") AND NOT KEY ("child, preschool") AND NOT KEY ("preschool child") AND (LIMIT-TO (SRCTYPE, "j")) AND (LIMIT-TO (LANGUAGE,"English")) AND (LIMIT-TO (DOCTYPE, "ar"))
Cochrane	Title Abstract Keyword ("magnetic resonance" OR MRI OR MR OR "amide proton transfer" OR "chemical exchange saturation transfer" OR "diffusion-weighted imaging" OR "diffusion tensor imaging" OR "susceptibility-weighted imaging" OR "quantitative susceptibility mapping" OR "diffusion kurtosis imaging" OR "perfusion-weighted imaging" OR "arterial spin labelling") AND Title Abstract Keyword (glioblastoma OR GBM OR "high-grade glioma")
EBSCO (Academic Search Ultimate)	AB (("magnetic resonance" OR MRI OR MR OR "amide proton transfer" OR "chemical exchange saturation transfer" OR "diffusion-weighted ima*" OR "diffusion tensor ima*" OR "susceptibility-weighted ima*" OR "quantitative susceptibility map*" OR "diffusion kurtosis ima*" OR "perfusion-weighted ima*" OR "arterial spin labe*") AND (glioblastoma OR GBM OR "high-grade glioma")) OR KW (("magnetic resonance" OR MRI OR MR OR "amide proton transfer" OR "chemical exchange saturation transfer" OR "diffusion-weighted ima*" OR "diffusion tensor ima*" OR "susceptibility-weighted ima*" OR "quantitative susceptibility map*" OR "diffusion kurtosis ima*" OR "perfusion-weighted ima*" OR "arterial spin labe*") AND (glioblastoma OR GBM OR "high-grade glioma"))
Embase (Ovid)	Abstract ("magnetic resonance" OR MRI OR "amide proton transfer" OR "chemical exchange saturation transfer" OR "diffusion-weighted imag*" OR "diffusion tensor imag*" OR "susceptibility-weighted imag*" OR "quantitative susceptibility map*" OR "diffusion kurtosis imag*" OR "perfusion-weighted imag*" OR "arterial spin label*") AND (glioblastoma OR GBM OR "high-grade glioma") OR Title ("magnetic resonance" OR MRI OR MR OR "amide proton transfer" OR "chemical exchange saturation transfer" OR "diffusion-weighted imag*" OR "diffusion tensor imag*" OR "susceptibility-weighted imag*" OR "quantitative susceptibility map*" OR "diffusion kurtosis imag*" OR "perfusion-weighted imag*" OR "arterial spin label*") AND (glioblastoma OR GBM OR "high-grade glioma") OR Keyword Heading ("magnetic resonance" OR MRI OR MR OR "amide proton transfer" OR "chemical exchange saturation transfer" OR "diffusion-weighted imag*" OR "diffusion tensor imag*" OR "susceptibility-weighted imag*" OR "quantitative susceptibility map*" OR "diffusion kurtosis imag*" OR "perfusion-weighted imag*" OR "arterial spin label*") AND (glioblastoma OR GBM OR "high-grade glioma")

The search terms were selected in order to capture all major advanced MRI modalities, accounting for the disparate naming conventions for identical technologies both over time and between locales. The major modalities include chemical exchange saturation transfer (CEST), DWI, PWI, SWI and MRS – these are comprised of modality-specific techniques and acquisition sequences that produce differing, nuanced images. These terms were refined through an iterative process involving consultation with a research liaison librarian and pilot searches in PubMed.

Database-specific filters will be utilised to isolate primary sources of evidence pertaining to adult patients that were published in English, notably, the publication dates of the search will be left unrestricted. The complete details regarding the search terms, search limitations, database-specific syntax, and filters to be applied to each database are listed in Table 1.

### Eligibility criteria

The criteria for evaluating the eligibility of papers for inclusion were developed by the authors, ensuring that the focus of the review remained on the utility of advanced MRI techniques for the preoperative imaging of glioblastoma patients. The following inclusion and exclusion criteria were defined and will be enforced: the population salient to this review will be adult glioblastoma patients with a newly diagnosed, untreated primary tumour confirmed not to harbour an IDH mutation. This restriction ensures any synthesis aligns with the 2021 WHO classification, which defines IDH-wildtype glioblastoma as the canonical tumour type, enhancing clinical relevance for utilisation in the preoperative setting. Low-grade gliomas and paediatric gliomas differ substantially from adult glioblastoma in molecular profile, growth patterns, treatment paradigms and prognosis, and hence will be excluded to preserve the focus on the defined target population, thereby honing the information gained in this synthesis to a more homogenous cohort, particularly in the context of the 2021 WHO revision to the classification system. Studies investigating mixed cohorts, such as high-grade gliomas more generally or longitudinal investigations including recurrent glioblastoma, that also report the preoperative, primary glioblastoma-specific results will be included, meanwhile, those that do not provide results from this subgroup will be excluded. This will reduce confounds and facilitate more accurate conclusions to be drawn regarding what advanced MRI may offer, while also ensuring a higher clinical relevance. To this end, studies using MRI to assess novel therapies, therapeutic targets, or other non-surgical interventions in a clinical trial will be excluded to maintain the homogeneity of the preoperative and untreated cohort of patients, and thereby facilitate a consistent radiologic characterisation of glioblastoma.

Studies using non-hydrogen-based MRI methods (e.g. carbon-13 and phosphorous-31 based magnetic resonance spectroscopy) will be excluded, as will those reporting only conventional MRI results, namely the structural imaging sequences (T1-weighted, T2-weighted, T2-fluid-attenuated inversion recovery). Additionally, studies that employed non-standard or experimental exogenous contrast agents (non-gadolinium-based), functional MRI without blood-oxygen level dependent signal reporting, or diffusion MRI focussed solely on tractography and connectivity will not be considered.

Only primary sources of evidence, available in English, from peer-reviewed journals that provided results derived from a sample size of at least five glioblastoma patients are eligible, subject to full-text availability; hence, reviews, case reports, meta-analyses, method papers, technical reports, preprints and conference proceedings will also be excluded.

Included articles will need to provide both MRI-derived metrics and a biological insight, such as correlations with markers of cell proliferation (Ki-67 or MIB-1), microvascular proliferation (CD31) or immune response (PD-L1 or CD68), or associations with the methylation of the MGMTp, the amplification of EGFR, or the mutation of the TERTp. Furthermore, articles relying on machine-learning (ML), artificial intelligence (AI), neural networks, regression models or radiomics will be excluded. This exclusion is not to dismiss ML/AI, but to bound the scope of this review, which prioritises direct imaging–biology relationships over predictive modelling approaches. It also serves to not reproduce existing reviews that address the application of specific models to various advanced MRI analyses [[32], [33], [34], [35], [36], [37], [38], [39], [40]]. We acknowledge that this decision may omit emerging methodologies that aim to link imaging features to tumour biology through interpretable models. In time, future reviews could be conducted to specifically focus on the continued developments in ML/AI and radiomics approaches, which represent an important and rapidly evolving area of research that will require a new, dedicated synthesis.

### Selection of sources of evidence

The results of this search strategy will be uploaded to the screening tool Covidence (Covidence systematic review software, Veritas Health Innovation, Melbourne, Australia. Available at www.covidence.org), a web-based collaboration software platform that streamlines the production of systematic and other literature reviews and removes duplicates. Eligibility criteria and exclusion reasons will be pre-configured in Covidence, aligned with the PCC framework. The platform will also be used to highlight key words and standardise reasons for the exclusion of full texts. Two independent reviewers will conduct the initial title and abstract screening, followed by a more in-depth full text review, in accordance with the criteria described above. Any discrepancies will be resolved through discussion or, if necessary, adjudicated by a third reviewer. The number of results at each stage of this protocol will be depicted in a PRISMA flow diagram.

### Data extraction

Studies that pass the screening process will be exported to EndNote for manual data extraction. The extraction and charting of the data will be performed by a single reviewer using a data extraction matrix created in Microsoft Word. A second reviewer will independently verify the data extraction process for a subset (approximately 30%) of eligible records to help ensure the reproducibility and reliability, as well as reduce the bias generated from a single reviewer during this stage. The variables to be extracted were determined by both the research question and reference to similar scoping reviews investigating other pathologies. However, due to the broad nature of the question, the matrix contains a mix of specific and generalised headings which facilitates an effective scoping of this research topic. Any inconsistencies that arise will be discussed between co-authors, however, no process for obtaining or confirming data from investigators will be employed for the purpose of this review. A draft of the data extraction matrix is provided in Appendix A; any modifications made will be detailed in the complete scoping review. The verification process will include cross-checking of key variables (MRI parameters, biological markers, sample characteristics, and main findings) with discrepancies resolved through discussion and, if necessary, consultation with a third reviewer. Inter-rater reliability will be assessed using percentage agreement for both the screening stage and the subset verification. Any systematic discrepancies identified during verification will trigger re-extraction of affected variables across all studies. Protocol adherence will be validated through regular team meetings to review progress, discuss emerging challenges, and document any necessary deviations from the planned methodology. Any protocol modifications will be transparently reported in the final review with justification for the changes.

The following information will be extracted from each included study:

• Article characteristics: year of publication and country of origin,
• MRI parameters: modality, technique, sequence, derived metrics, magnetic field strength (B_0_), repetition time (TR), and echo time (TE),
• Patient population: different types of brain tumours or other pathologies mentioned, mutations present, sex distribution, mean and standard deviation of age, and sample size,
• Study design: prospective or retrospective nature, and study aims,
• Methodology: image processing techniques, analytical tools and approaches,
• Key findings: clinical relevance, biological insights into glioblastoma, reported limitations, future research suggestions, and summary points.

Notably, while the populations of interest for each study will be recorded, only the results pertaining to the preoperative, primary, IDH-wildtype glioblastoma cohort will be extracted for synthesis. Furthermore, to assist with the interpretation of the robustness of imaging-biology associations and succinctly assess the reliability of evidence, the following parameters will be reported alongside the findings: sample size, retrospective vs prospective study design, MRI field strength and the histopathological or molecular standard referenced for comparison/correlation with imaging.

This review focuses on IDH-wildtype glioblastoma to align the 2021 WHO reclassification of CNS tumours to maintain consistency with current diagnostic standards, thus it mandates that an assumption be made regarding the reclassification, that being that studies prior to 2021 that report on de novo and/or primary glioblastoma with an unreported IDH mutation status are equivalent to the current diagnostic criteria for glioblastoma, however, since the IDH mutation status became clinically relevant in the 2016 WHO classification, most recent studies explicitly report this marker. Historically, approximately 90% of all glioblastomas were classified as “primary” or “de novo,” which aligns closely with the prevalence of IDH-wildtype glioblastoma reported in large cohort studies using the current WHO classification [[45], [46], [47]]. This relationship is further supported by the Cancer Genome Atlas’ (TCGA’s) comprehensive genomic analysis of diffuse lower-grade gliomas, which demonstrated that IDH-wildtype tumours—even when histologically lower grade—share molecular hallmarks and clinical behaviour with primary glioblastoma, reinforcing the concept that IDH-wildtype glioblastoma corresponds to the historical primary glioblastoma category [36]. Conversely, IDH-mutant astrocytomas, represent the lineage that historically progressed to secondary glioblastoma, now classified as astrocytoma, IDH-mutant, WHO grade 4 [46]. Studies with inferable or reported IDH-wildtype glioblastomas will therefore be considered in this review accordingly. Conversely, ‘secondary glioblastoma’, which aligns with WHO grade 4 astrocytoma, IDH-mutant, will be excluded. Additionally, historical diagnoses of low-grade gliomas, which under current criteria may be reclassified as glioblastoma, will not be reinterpreted or included. This assumption introduces some risk of misclassification; therefore, studies will be stratified by known versus inferred IDH mutation status in the synthesis.

### Data analysis and synthesis of results

Included studies will be synthesised according to a structured framework that groups them by the biological domain (molecular factors, microenvironmental, vascular, metabolic, immune response) they report on, with subgroups defined by the MRI modalities (or combination of such) that they utilised; studies utilising the same combinations of MRI modalities will also be grouped together accordingly, as will the reports of their findings. Within these groups, relevant MRI-derived metrics and key findings will be summarised, with additional notes made regarding the sample size of the study cohort, the aim of the research and whether the IDH-wildtype status was reported or inferred. To ensure that the findings reported from eligible articles are not misconstrued, the method of IDH-wildtype status ascertainment will be clearly stated throughout the mapping of evidence and synthesis of results. This will help findings from inferred/unclear cohorts to not be conflated or interpreted equivalently to molecularly confirmed IDH-wildtype cohorts. For each biological domain and MRI modality combination, studies will be grouped by methodological similarity, and patterns of convergence or divergence in findings will be explicitly noted. Sources of heterogeneity will be systematically documented, including MRI field strength, sequences, region of interest definition methods, sample sizes, patient demographics, and analytical approaches. When conflicting results are identified, potential explanations will be explored by examining differences in study design, technical parameters, and patient populations. Uncertainty will be addressed by documenting the consistency of findings across studies, noting when single studies report isolated findings, and highlighting areas where evidence is limited or contradictory. This structured approach to heterogeneity assessment will enable the review to distinguish between genuine evidence gaps, methodological inconsistencies, and robust findings.

When studies report metrics, we will adopt the original form without transformation. Units will be stated, and rounding will never exceed the original study’s precision. We will not compute new p‑values or CIs. Statistical significance will be shown exactly as reported. Reported correlations will be retained as published and interpreted narratively. We will assess whether each reported association rests primarily on single‑centre correlation without corroboration, has small samples, lacks multiplicity control or confidence intervals, or shows sensitivity to analytic choices that undermines interpretability.

The synthesis will be presented in multiple formats, as appropriate to the content of the information being conveyed. This includes charts that illustrate the frequency of MRI modality usage, the prevalence of multi-modal MRI approaches, the distribution of study designs, and cohort sizes. These visualisations will support conclusions addressing the central question underlying this review, “can advanced MRI help understand the biology of glioblastoma?”, by answering the six RQs specified prior. It will also serve to identify and classify knowledge gaps that are present in the field, by systematically assessing areas where evidence is sparse, inconsistent, or absent within each biological domain and MRI modality. These may be categorised as: (1) underrepresented biological domains, (2) limited modality coverage, (3) methodological limitations, and (4) unresolved clinical correlations.

### Expected outcomes

This review will produce a structured evidence map of how advanced MRI metrics relate to key biological domains in adult, preoperative, IDH‑wildtype glioblastoma, contextualising findings by documenting methodological heterogeneity and uncertainty as recommended in scoping‑review guidance (PRISMA‑ScR; JBI). Concretely, we will deliver: a narrative synthesis that preserves the source studies’ statistics and wording and notes the comparability and uncertainty for and between each study. We will also provide a summary of areas where evidence is consistent versus exploratory. These outputs are intended to clarify what is known, where uncertainty originates, and where additional evidence is required. This is designed to inform future experimental design by collating which protocols and post‑processing practices recur in studies reporting biologically consistent associations, aiding with design choices that could be prioritised in prospective work or multi‑centre trials aiming to improve harmonisation and reproducibility. Furthermore, the review will support clinical translation and standardisation efforts by putting forward candidate imaging biomarkers and by summarising the degree to which current literature shows utility. This will help investigators, to design pragmatic harmonisation plans for multi‑site studies.

### Data availability and transparency

To ensure reproducibility and enable independent verification of our findings, we commit to open science practices. Upon completion of this scoping review, we will deposit the following materials in an open-access repository (e.g. OSF, Figshare [https://figshare.com/], or an institutional repository):

1. The complete extracted database in MS-Excel format, including all variables specified in the data extraction matrix (Appendix A).
2. The final data extraction template (including any modifications made during implementation).
3. Screening decisions and exclusion rationales at both title/abstract and full-text stages, exported from Covidence with documented reasons.
4. Any scripts, tools, or code used for data synthesis, visualisation, or analysis.

All materials will be assigned persistent identifiers (DOIs) and referenced in the final publication to enable independent verification, reanalysis, and extension of our findings. This commitment aligns with contemporary standards for reproducible research and addresses the scientific community's responsibility to ensure research integrity.

## Protocol validation

This scoping review protocol was developed in accordance with the PRISMA-ScR framework [41] and JBI guidance [42–44,48] to ensure the feasibility and transparency of the review from its initial ideation. It will also undergo further testing and validation by being implemented in a trial run of the searches, which will also serve to verify the accurate translation of syntax and Boolean operators between database-specific queries. The eligibility criteria and the data extraction matrix will also be piloted to ensure they are fit for purpose and effectively capture data relevant to the research question.

### Preliminary yield

A preliminary implementation of the finalised search strategy for PubMed alone on 28 October 2025 retrieved 3091 records. This confirmed that the strategy captures studies across the intended broad scope while remaining manageable, noting that a considerable number of duplicates are expected by searching the selected five databases, which are easily/readily removed, leaving only the unique records for screening.

## Limitations

This scoping review protocol has several limitations. It excludes grey literature (unpublished and non-traditional research outputs), which may result in the omission of early or emerging data that could provide valuable insights into novel imaging approaches or biological associations. This exclusion was primarily driven by feasibility constraints and the need to ensure methodological consistency, as grey literature often lacks peer review and may introduce uncertainty regarding data quality. While supplementary searches or sensitivity analyses could partially mitigate this limitation, these methods were not adopted due to resource constraints and the exploratory nature of this review. Consequently, the comprehensiveness of the evidence base may be reduced.

This review is also restricted to articles published in English, which may limit the comprehensiveness and global representation of findings, introducing potential language and geographic biases. This restriction was necessary to ensure feasibility and accuracy in screening and data extraction, as translation of non-English studies would require substantial resources and could introduce additional errors. This could lead to an underrepresentation of findings in non-English speaking regions which may differ in patient demographics or molecular profiles. While this restriction is common to increase feasibility, it should be acknowledged that it may reduce the global applicability of the review’s findings.

Furthermore, the wide search strategy is expected to yield a high volume of results, the majority of which may be redundant (duplicates or irrelevant). However, this approach is intended to maximise sensitivity and help ensure that all relevant studies are captured through manual screening. Moreover, we define biological insight broadly to capture the full potential of advanced MRI for relating to glioblastoma biology across study designs, modalities, and endpoints. An overly prescriptive framework risks excluding promising but differently framed work and could bias the resulting evidence map. Therefore, we adopt an inclusive, domain‑based definition at protocol stage and reserve any sharpening of theoretical claims for the completed synthesis. Additionally, since most data extraction will be undertaken by a single reviewer, there is potential for omission, miscoding, or subjective interpretation, which could distort the apparent consistency/divergence of findings or misclassify IDH ascertainment and technical parameters. To mitigate this, we have pilot‑calibrated the extraction form on a small set of diverse studies with team consensus and will apply second‑reviewer verification to a stratified ∼30% subset, documenting discrepancies and their resolution. These measures reduce, but do not eliminate, bias; consequently, our synthesis remains descriptive and any statements of consistency or divergence are cautious. This approach accords with PRISMA‑ScR/JBI guidance for scoping reviews to emphasise transparent charting, documentation of heterogeneity and uncertainty, and cautious interpretation rather than new inferential analyses. Moreover, as a scoping review methodology was chosen, we will not pool estimates or assign new significance; numerical precision and statistical significance will be reproduced from source studies and presented with modality‑appropriate rounding.

Moreover, this scoping review protocol operates on the assumption that studies published prior to the 2021 WHO classification that report on de novo or primary glioblastoma without specifying IDH mutation status are equivalent to IDH-wildtype glioblastoma. While this assumption is pragmatic and necessary for consistency, it introduces a risk of misclassification that may affect the accuracy of biological interpretations – however, due to the exploratory nature of the planned scoping review, the magnitude of this impact is predicted to be minimal.

Lastly, the exclusion of studies employing ML, AI and radiomics may limit the review’s ability to capture the analytical approaches that are increasingly used in neuro-oncologic imaging. The scientific implications of this exclusion include potential underestimation of the full predictive capacity of advanced MRI when combined with advanced computational approaches, and the review may not capture emerging hybrid methods that combine traditional imaging metrics with machine learning for biological inference. This exclusion is believed to be a strength of the protocol as it pragmatically bounds the scope of the review, allowing it to provide a focussed synthesis of direct imaging-biology relationships. However, this exclusion may bias the scope by omitting emerging methodologies that aim to link imaging features to molecular markers through interpretable models. Nevertheless, this focused scope enables deeper examination of direct, interpretable imaging-biology relationships that can inform mechanistic understanding and hypothesis generation for future validation studies.

## CRediT author statement

**James Brown-Miles:** conceptualisation, methodology, validation, formal analysis, investigation, resources, data curation, writing – original draft, writing – review and editing, visualisation, project administration. **Oun Al-iedani:** methodology, writing – review and editing, supervision. **Peter Greer:** writing – review and editing, supervision. **Michael Fay:** writing – review and editing, supervision. **Hubert Hondermarck:** conceptualisation, writing – review and editing, supervision. **Saadallah Ramadan:** conceptualisation, methodology, writing – review and editing, supervision.

## Related research article

None.

## Declaration of competing interest

The authors declare that they have no known competing financial interests or personal relationships that could have appeared to influence the work reported in this paper.

## Appendix Supplementary materials

Supplementary material *and/or* additional information [OPTIONAL] Supplementary material associated with this article can be found in the online version. Image, application 1
