# Supplementary material for: Exploring the role of advanced MRI in understanding glioblastoma biology: A scoping review protocol
Source: MethodsX. 2026 Jun 2;16:103984. doi: 10.1016/j.mex.2026.103984 (PMC13251193; doi:10.1016/j.mex.2026.103984)
Supplement: Supplementary file 1 — Supplementary material and/or additional information [OPTIONAL] Supplementary material associated with this article can be found in the online version. [file mmc1.docx]

| Year | First Author | Title | Country | MRI Modality | MRI Technique | MRI Sequences | MRI Metrics | B0 / TR / TE | Post-Processing Method | Analysis Method | Clinical Aspect | Relation to Biology | Limitations | Suggestions | Known Mutations | Sample Size | Sex / Mean Age | Study Design | Aims | Key Points |
| --- | --- | --- | --- | --- | --- | --- | --- | --- | --- | --- | --- | --- | --- | --- | --- | --- | --- | --- | --- | --- |
|  |  |  |  |  |  |  |  |  |  |  |  |  |  |  |  |  |  |  |  |  |

*Table 1 - Data extraction matrix for eligible articles. Abbreviations: MRI, magnetic resonance imaging; B0, static magnetic field strength; TR, repetition time; TE, echo time.*
